# Asteroid Hyalosis Seen on Ocular Point-of-Care Ultrasound

**DOI:** 10.5811/cpcem.2019.6.42935

**Published:** 2019-07-22

**Authors:** Blake Arthurs, Randi Connor-Schuler, Wendelin Kreifels, Julian Suszanski, Sudhir Baliga, David Amponsah

**Affiliations:** * Wayne State University School of Medicine, Detroit, Michigan; † Henry Ford Hospital, Department of Emergency Medicine, Detroit, Michigan

## Abstract

We present a case of a patient who underwent ultrasound evaluation for potential blunt ocular trauma. She was found to have multiple, freely mobile, scintillating hyperechoic opacities within the vitreous that was diagnosed as asteroid hyalosis, a rare but benign condition easily confused with vitreous hemorrhage, retinal detachment, lens dislocation, or foreign body on ocular ultrasound.

## CASE PRESENTATION

**Image 1:**
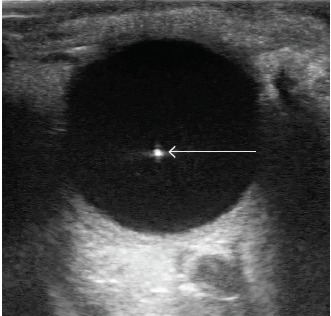
B-mode sonogram showing an echogenic particle (arrow) floating within the body of the vitreous in a patient with asteroid hyalosis.

**Image 2:**
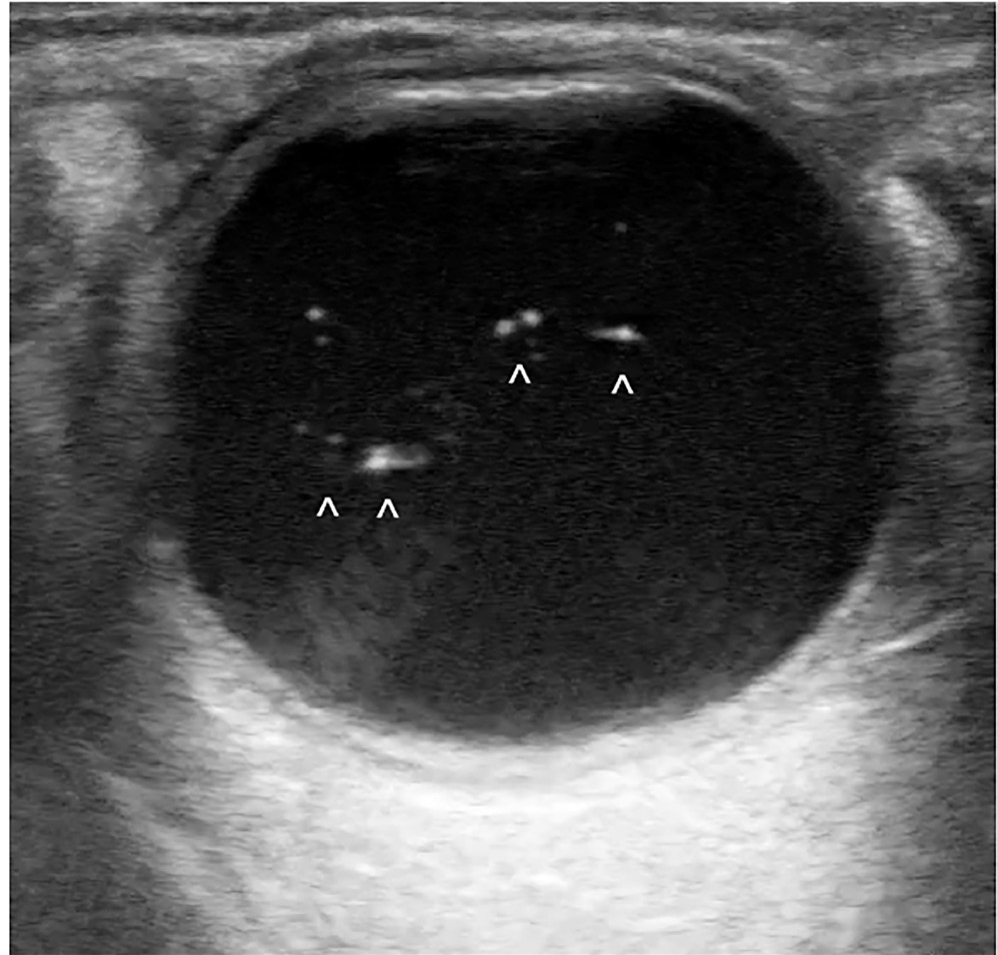
B-mode sonogram demonstrating several discrete, free-floating, echogenic particles (^) consistent with asteroid hyalosis.

An 86-year-old woman with a history of hyperlipidemia presented to the emergency department after a fall. Exam was significant for altered mental status and signs of head trauma with left periorbital swelling and ecchymosis. Ocular ultrasound was performed using an ocular preset and with a Sonosite 10-MHz linear transducer to further assess for potential traumatic eye pathology. Ultrasound of the left eye was unremarkable, however the right eye demonstrated discrete, freely mobile, scintillating, hyperechoic opacities scattered throughout the vitreous (Images 1 and 2; Video).

## DIAGNOSIS

Asteroid hyalosis (AH) is a benign, degenerative condition of fatty calcium soap deposits in the vitreous that usually occurs unilaterally and typically occurs in men ages 75–86.^1^–^4^ Previous research has shown that these spherical asteroid bodies are composed of calcium and phosphate complexed with lipids and are usually asymptomatic.^1^,^5^ Asteroid bodies can be visualized by ophthalmoscope examination as well as B-mode ultrasonography, as this case demonstrates, with a shimmering, starry-night appearance to the vitreous. While rare, it is important to be aware of AH as it is a benign condition that can be confused with other ocular pathologies typically seen on ultrasound, including vitreous hemorrhage, foreign body, and retinal detachment.

AH can be most easily confused with vitreous hemorrhage.^6^,^7^ While vitreous hemorrhage may have an echogenic appearance, particularly when the gain is increased, it will also have a fluid, heterogenous appearance that ebbs and flows with eye movements and typically layers posterior in the eye. In contrast, AH will have discrete, scintillating particles of more variable echogenicity scattered throughout the vitreous.^7^ It is important to differentiate the two conditions in the point-of-care setting as vitreous hemorrhage is often an acute pathology while AH is a benign condition that does not require urgent ophthalmology consultation or evaluation.

KEY ISSUES

- Point-of-care ocular ultrasound is performed using a high-frequency linear probe.
- Point-of-care ultrasound can be used to diagnose ocular disorders such as lens dislocation, foreign body, retinal detachment, and vitreous hemorrhage.
- AH can be confused with vitreous hemorrhage as both involve echogenic debris floating in the vitreous.
- Vitreous hemorrhage has an “oil in water” appearance with more fluid echogenic material that will ebb and flow with ocular movements, in contrast to AH, which involves individual echogenic particles that will undergo small movements in the vitreous with ocular movement.
- It is important to differentiate between vitreous hemorrhage and AH given the clinical implications. While vitreous hemorrhage is pathologic and requires ophthalmology consultation, AH is a benign condition that does not require ophthalmologic evaluation.

**CPC-EM Capsule**

What do we already know about this clinical entity?

Asteroid hyalosis (AH) is a benign condition more familiar to ophthalmologists and less well known to emergency physicians.

What is the major impact of the image(s)?

These images highlight the appearance of AH on ultrasound with scintillating echogenic particles seen within the vitreous body vs alternative diagnoses such as vitreous hemorrhage.

How might this improve emergency medicine practice?

With the increasing use of point-of-care ultrasound, it is important to distinguish normal, benign conditions from pathology that requires specialist intervention.

## Supplementary Information

Video. B-mode ultrasound clip showing multiple scintillating hyperechoic foci consistent with a diagnosis of asteroid hyalosis.
